# Profiles of SUMO and ubiquitin conjugation in an Alzheimer's disease model

**DOI:** 10.1016/j.neulet.2011.07.045

**Published:** 2011-09-20

**Authors:** Laura E. McMillan, Jon T. Brown, Jeremy M. Henley, Helena Cimarosti

**Affiliations:** a MRC Centre for Synaptic Plasticity, School of Biochemistry, Medical Sciences Building, University of Bristol, University Walk, Bristol BS8 1TD, UK; b MRC Centre for Synaptic Plasticity, School of Physiology and Pharmacology, Medical Sciences Building, University of Bristol, University Walk, Bristol BS8 1TD, UK; c School of Pharmacy, Hopkins Building, University of Reading, Whiteknights, Reading RG6 6UB, UK

**Keywords:** Alzheimer's disease, Glutamate receptors, GluA1, GluK2/3, Posttranslational modification, SENP-1, SUMO, Tg2576 mice, UBC9, Ubiquitin

## Abstract

Alzheimer's disease (AD) is a major cause of disability in the elderly. The formation of senile plaques and neurofibrillary tangles are the main hallmarks of the disorder, whereas synaptic loss best correlates to the progressive cognitive decline. Interestingly, some of the proteins involved in these pathophysiological processes have been reported to be subject to posttranslational modification by ubiquitin and/or the small ubiquitin-like modifier (SUMO). Here we investigated global changes in protein SUMOylation and ubiquitination *in vivo* in a model of AD. We used Tg2576 transgenic mice, which overexpress a mutated human amyloid precursor protein (APP) gene implicated in familial AD. As expected, APP protein levels were dramatically increased in the hippocampus, cortex and cerebellum of Tg2576 mice. A significant increase in the global level of ubiquitinated proteins was observed in the hippocampus of Tg2576 mice. Significant or close to significant changes in individual bands of SUMO-1 or SUMO-2/3 conjugation were apparent in all brain regions investigated, although global levels were unaltered between wild-type and transgenic mice. Levels of SUMO-specific conjugating and deconjugating enzymes, UBC9 and SENP-1 were also unaltered in any of the brain regions analysed. Surprisingly, given the well-documented loss of synaptic function, total levels of the excitatory AMPA and kainate receptors were unaffected in the Tg2576 mice. These results suggest that alterations in SUMO substrate conjugation may occur and that global posttranslational modifications by ubiquitin may play an important role in the mechanisms underlying AD.

## Highlights

► Global levels of ubiquitinated proteins increased in the hippocampus of Tg2576 mice. ► No global changes in either SUMO-1 or SUMO-2/3 conjugation in any brain regions analysed. ► SUMO conjugating and deconjugating enzymes, UBC9 and SENP-1, unaltered in Tg2576 mice. ► Total levels of AMPA and kainate receptors were also unaffected in Tg2576 mice. ► Posttranslational modification by ubiquitin may play a role in Alzheimer's disease.

Alzheimer's disease (AD) is the most common neurodegenerative disorder and cause of dementia among the aging population (http://www.alz.co.uk/research/worldreport/). It has a complex pathophysiology, which, although not completely understood, is characterised by beta-amyloid (Aβ) senile plaques originating from cleavage of the amyloid precursor protein (APP) and neurofibrillary tangles formed from hyperphosphorylated microtubule protein tau [28]. Synaptic loss is another characteristic feature of the condition and probably the best correlate of the cognitive decline that develops progressively in AD patients [27]. Several lines of evidence indicate that excitatory synapses are lost due to a decrease in AMPA and NMDA receptors at the cell surface caused by extracellular accumulation of Aβ [24].

Ubiquitination and SUMOylation are key protein modification pathways that involve a sequence of analogous but distinct enzymatic steps to covalently conjugate either ubiquitin or SUMO (small ubiquitin-like modifier) to substrate proteins. Generally, ubiquitinated proteins are targeted to the proteasome for degradation (reviewed in [25]), whereas proteins tagged by one of the validated SUMO paralogues (SUMO-1 to -3, the difference between SUMO-2 and -3 being only three amino acids) can undergo changes in localisation, activity and/or stability (reviewed in [29]). Both ubiquitin and SUMO conjugation pathways play critical roles in diverse physiological and pathophysiological processes [22,30,32]. High levels of ubiquitinated proteins have been observed in all major neurodegenerative disorders including AD [18]. Recently, alterations in global levels of SUMOylated proteins have been reported in a number of these diseases (for review see [4]) and several AD-associated proteins, including APP and tau, have been shown to be SUMOylated [9,11,33].

Tg2576 transgenic mice, which overexpress a mutated APP gene containing the Swedish familial AD mutation, are a widely recognised model of AD [14]. These mice exhibit elevated Aβ that lead to plaque pathology and behavioral deficits analogous to that of AD patients (for review see [14]). Previous *in vivo* and *in vitro* studies using Tg2576 mice have shown impairment of the ubiquitin–proteasome system as well as reduction in the surface expression of AMPA receptors [2,3,23]. Here we investigated potential changes in protein SUMOylation and ubiquitination patterns, SUMO-specific conjugating (UBC9) and deconjugating (SENP-1) enzymes, and levels of AMPA and kainate (KA) receptor subunits in Tg2576 mice. As expected, our results show a dramatic increase of APP protein levels in the hippocampus, cortex and cerebellum, though surprisingly, given the reported loss of synaptic function, levels of the excitatory AMPA and KA receptors were unaffected. A robust increase in total ubiquitinated proteins was seen in the hippocampus of Tg2576 mice, but not in the cortex or cerebellum. A decrease in SUMO-2/3 conjugation of high molecular weight proteins was evident in the cortex of transgenic mice, and although changes in the modification of multiple SUMO-1 and SUMO-2/3 conjugates were apparent in all brain regions, no other significant changes in conjugation or in levels of SUMO conjugation machinery were observed between transgenic and control mice.

Animal care and experimental procedures were conducted in accordance with UK Home Office legislation and experimental protocols approved by the British National Committee for Ethics in Animal Research.

Hippocampal, cortical and cerebellar regions were prepared from 9-month male Tg2576 mice and wild-type age-matched controls processed in parallel. Brains were immediately removed following cervical dislocation and dissected in ice cold Hank's balanced salt solution (HBSS, pH 7.2) containing (in mM): 1.26 CaCl_2_, 5.36 KCl, 136.89 NaCl, 36.08 glucose, 0.44 KH_2_PO_4_, 0.34 Na_2_HPO_4_, 0.49 MgCl_2_, 0.44 MgSO_4_, 25 HEPES and 4 NaHCO_3_. For each of the regions, both hemispheres were pooled together in ice-cold lysis buffer containing: 50 mM Tris–HCl (pH 7.4), 150 mM NaCl, 1 mM EDTA, 0.1% SDS, 1% Triton X-100, 1% mammalian protease inhibitor (Roche) and 20 mM NEM (Sigma–Aldrich). For the tissue homogenisation protocol, equivalent amounts of tissue were lysed in equivalent buffer volumes, samples were sonicated for 10 s at 4 °C and total protein concentrations were determined as previously reported [8].

An appropriate volume of loading buffer consisting of 125 mM Tris–HCl (pH 6.8), 20% glycerol, 4% SDS, 0.01% bromophenol blue and 5% β-mercaptoethanol was added to the samples, which were then boiled for 5 min at 95 °C. Equal amounts of protein loaded at 10–50 μg protein/lane were resolved by SDS-PAGE (8–15%) and immunoblotting was performed as formerly described [8]. The primary antibodies used were: rabbit polyclonal anti-APP (Sigma; 1:4000), mouse monoclonal anti-ubiquitin (Santa Cruz; 1:500), rabbit polyclonal anti-SUMO-1 (1:4000) kindly provided by Dr. M. Dasso, rabbit polyclonal anti-SUMO-2/3 (Zymed; 1:250), rabbit polyclonal anti-SENP-1 (Imgenex; 1:1000), rabbit polyclonal anti-UBC9 (Santa Cruz; 1:250), mouse monoclonal anti-GluA1 (Millipore; 1:2000), rabbit monoclonal anti-GluK2/3 (Upstate; 1:1000) and mouse monoclonal anti-β-actin (Sigma; 1:10,000). For each region (hippocampus, cortex and cerebellum), samples from at least six Tg2576 mice were compared to an equal number of wild-type age-matched controls on the same Western blot to avoid differences in signal intensity. The same blots were then re-probed with anti-β-actin antibody as an internal control to ensure equal protein loading in all lanes. Densitometry was performed on blots for each brain region and analysed using Image J (NIH). For SUMO and ubiquitin conjugate multiple bands the entire lane with a maximum range of 15–250 kDa as well as individual prominent bands were analysed. Band intensity values for the investigated proteins were normalised against the internal loading control values and the mean normalised value obtained for the wild-type group was designated as 100%.

Results are expressed as mean ± SEM of the indicated number of animals. Student's *t*-test was applied to the means to determine differences between experimental groups. *p* Values < 0.05 were considered statistically significant.

**Fig. 1:**
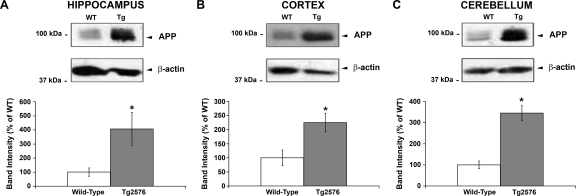
APP protein levels are increased in Tg2576 transgenic mice in all brain regions analysed. Nine-month Tg2576 transgenic and age-matched wild-type mice were decapitated, their brains removed and dissected into hippocampus, cortex and cerebellum. Regions from both hemispheres were pooled together and tissue lysates prepared for Western blotting. Representative immunoblots showing APP immunoreactivity and the respective β-actin loading controls in the hippocampal (A), cortical (B) and cerebellar (C) regions of wild-type and Tg2576 mice. The quantified APP data shown for each region was obtained from at least six animals of each group. The results are presented as percentage of wild-type ± SEM. *Significant difference compared to wild-type, *p* < 0.05.

Tg2576 mice show increased APP protein levels in all brain regions analysed: To confirm that Tg2576 mice, which overexpress the Swedish double mutation (K670N, M671L) of the human APP gene, produce increased levels of APP protein compared to their age-matched controls, we performed Western blots in samples from brain regions of transgenic and wild-type mice. As expected, the transgenic mice express increased APP protein levels by at least 3-fold in the hippocampus (Fig. 1A), cortex (Fig. 1B) and cerebellum (Fig. 1C). The increase in APP protein levels in the transgenic mice provides increased substrate for β-secretase, an enzyme that proteolytically cleaves APP to produce Aβ, increased levels of which, in turn, lead to amyloid plaque accumulation in this mouse model (for reviews see [12]).

**Fig. 2:**
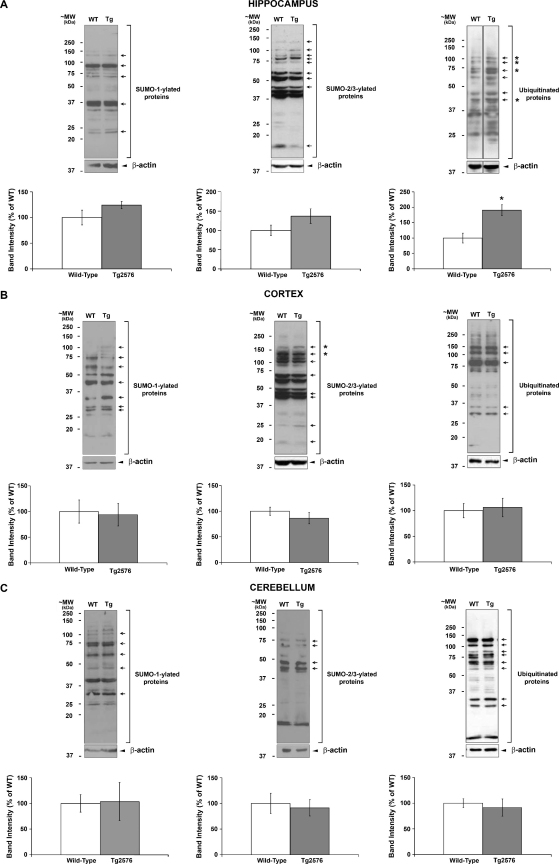
Pattern of global protein SUMOylation and ubiquitination in Tg2576 transgenic and age-matched wild-type mice. Representative immunoblots of global SUMO-1 (left panel), SUMO-2/3 (center panel) and ubiquitin (right panel) conjugated proteins and their respective β-actin loading controls in the hippocampus (A), cortex (B) and cerebellum (C) of wild-type and Tg2576 mice. The experimental protocol was as described in Fig. 1. Cumulative global levels of SUMO-1-, SUMO-2/3- and ubiquitin-conjugates for each region showing quantified data from at least six animals of each group are presented as percentage of wild-type ± SEM. In addition, the arrows indicate prominent bands that were individually quantified and the presence of an * denotes a significant difference compared to wild-type, *p* < 0.05. The data from individual bands are presented in Table 1 and Supplementary Fig. S1.

Levels of protein SUMOylation and ubiquitination in Tg2576 mice: To determine if Tg2576 mice show modified SUMOylation and/or ubiquitination of substrate proteins, immunodetection using antibodies against SUMO-1, SUMO-2/3 and ubiquitin was performed in blots containing samples from Tg2576 and age-matched wild-type mice. The global levels and individual bands of posttranslationally modified proteins were analysed for hippocampus (Fig. 2A), cortex (Fig. 2B) and cerebellum (Fig. 2C).

Since global levels of SUMOylated proteins have been reported to be increased in a number of neurodegenerative diseases (for review see [4]) and key proteins in AD have been implicated as SUMO substrates or SUMO-interacting proteins [9,10,20,33], we hypothesised that altered levels of SUMO-conjugated substrates might be evident in Tg2576 mice. Conjugation by both SUMO-1 and SUMO-2/3 displayed similar global levels in both transgenic and age-matched wild-type mice in all of the brain regions analysed (Fig. 2A–C, left and centre panels). Similarly, the, global levels of ubiquitin conjugates in the cortex and cerebellar regions were analogous between transgenic and age-matched wild-type mice (Fig. 2B and C, right panels). In contrast, however, global levels of ubiquitin-conjugated proteins in the hippocampus of Tg2576 mice were significantly increased compared to age-matched wild-type mice (Fig. 2A, right panel).

**Supplementary Fig. S1:**
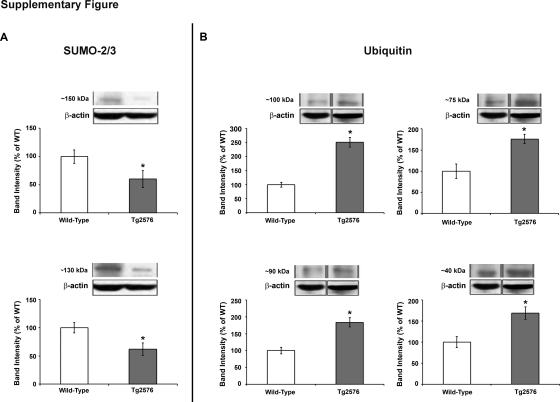
Changes in individual SUMO-2/3- and ubiquitin-conjugated proteins in Tg2576 transgenic compared to age-matched wild-type mice. Graphical presentation of the cumulative individual levels of SUMO-2/3-conjugates in cortex (A) and ubiquitin-conjugates in hippocampus (B). Representative bands and quantified data from at least six animals of each group are presented as percentage of wild-type ± SEM. The arrows in Fig. 2 indicate these prominent bands showed here, which were individually quantified and the presence of an * denotes a significant difference compared to wild-type, *p* < 0.05. These data is also presented in Table 1.

Analysis of a selection of prominent bands revealed significant or very close to significant changes in SUMO-1, SUMO-2/3 and ubiquitin conjugation levels of individual proteins (Table 1). In the hippocampus of Tg2576 mice, although increases in individual bands for SUMO-1 and SUMO-2/3 were apparent, these changes did not reach significance. Individual bands for SUMO-1 and SUMO-2/3 in the cortex show apparent decreases: most strikingly, two high molecular weight SUMO-2/3 conjugates at ∼130 and ∼150 kDa showed a significant decrease (Supplementary Fig. 1A). In the cerebellum, distinct bands in the SUMO-1 blot showed increases which were close to significance at ∼100 kDa and ∼60 kDa, whereas no other bands showed apparent alterations. In conjunction with the global changes in ubiquitin conjugation, several individual ubiquitin bands were significantly increased in the hippocampus of Tg2576 mice (Supplementary Fig. 1B). In contrast, no significant changes in ubiquitination were evident in the cortex and cerebellum.

**Fig. 3:**
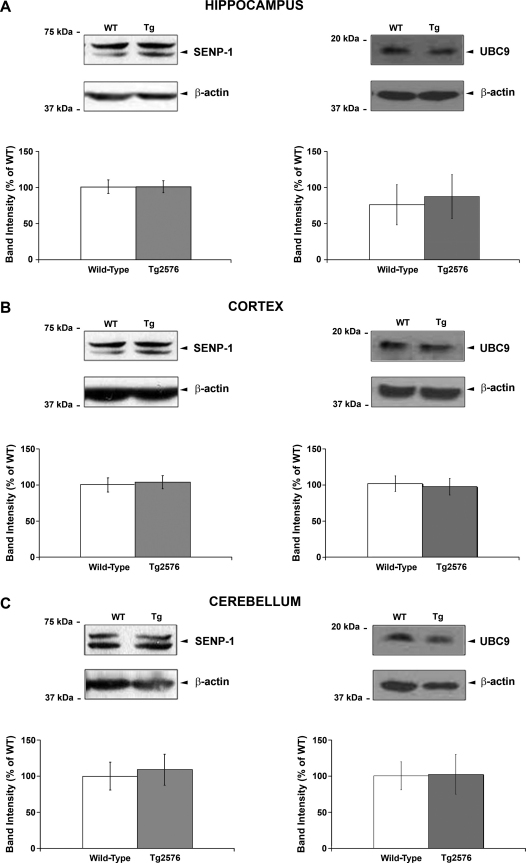
SENP-1 and UBC9 protein levels in Tg2576 transgenic and age-matched wild-type mice. Representative SENP-1 (left panels) and UBC9 (right panels) immunoreactivities and their respectives β-actin loading controls in hippocampus (A), cortex (B) and cerebellum (C) of wild-type and Tg2576 mice. The experimental protocol was as described in Fig. 1. Cumulative SENP-1 and UBC9 results for each region showing quantified data from at least six animals of each group are presented as percentage of wild-type ± SEM. No significant difference compared to wild-type, *p* > 0.05.

SUMO-specific conjugating and deconjugating enzymes protein levels in Tg2576 mice: To further investigate alterations in protein SUMOylation in this model, we analysed the protein levels of the SUMO conjugating enzyme, UBC9, and the SUMO-specific isopeptidase, SENP-1. Neither enzyme was significantly altered between the Tg2576 and age-matched wild-type mice (Fig. 3A–C).

**Fig. 4:**
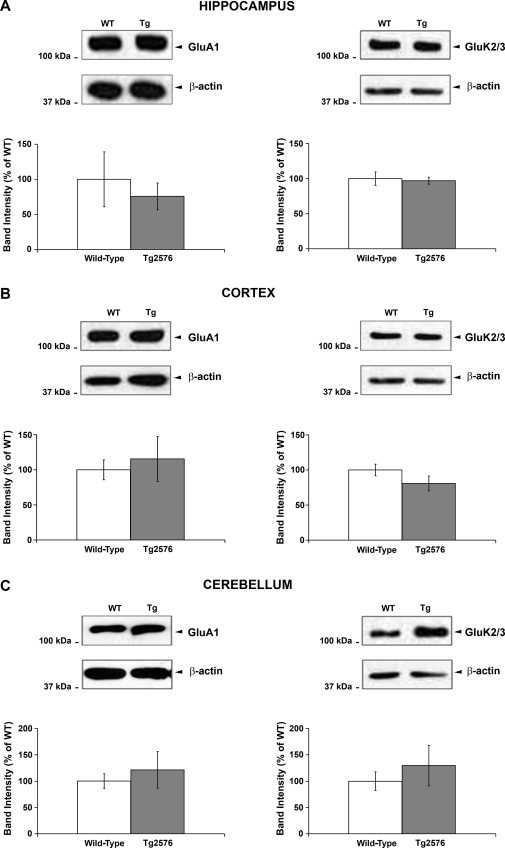
GluA1 and GluK2/3 total protein levels in Tg2576 transgenic and age-matched wild-type mice. Representative GluA1 (left panels) and GluK2/3 (right panels) immunoreactivities and their respectives β-actin loading controls in hippocampus (A), cortex (B) and cerebellum (C) of wild-type and Tg2576 mice. The experimental protocol was as described in Fig. 1. Cumulative GluA1 and GluK2/3 results for each region showing quantified data from at least six animals of each group are presented as percentage of wild-type ± SEM. No significant difference compared to wild-type, *p* > 0.05.

AMPA receptor subunit, GluA1, and KA receptor subunits, GluK2/3, protein levels in Tg2576 mice: Since synaptic dysfunction and/or loss is highly implicated as an early event in the pathophysiology of AD [24], we investigated total levels of the AMPA and KA receptor subunits, GluA1 and GluK2/3, respectively, in the Tg2576 mice using the same tissue samples monitored for ubiquitination and SUMOylation. The total levels of GluA1 and GluK2/3 were not altered in any of the brain regions analysed in Tg2576 compared to age-matched wild-type mice (Fig. 4A–C).

Our results confirm that APP protein levels are dramatically increased in the hippocampus, cortex and cerebellum of Tg2576 mice. Moreover, a significant increase in the global levels of ubiquitinated proteins, as well as several distinct molecular weight conjugates, was observed in the hippocampus of Tg2576 mice. Interestingly, significant decreases in distinct high molecular weight SUMO-2/3 conjugates were seen in the cortex of Tg2576 mice. Although multiple SUMO-1 and SUMO-2/3 bands appear to be altered in brain regions investigated, no other significant changes were detected. Levels of the SUMOylating, UBC9, and deSUMOylating, SENP-1, enzymes were also unaltered in any of the Tg2576 mice brain regions analysed. Surprisingly, given the reported loss of synaptic function [13,17], total levels of the excitatory AMPA and kainate neurotransmitter receptors were unaffected in the Tg2576 mice at the age we investigated.

Under pathological conditions, including AD, ubiquitin immunoreactive inclusion bodies accumulate, proteasomal activity is decreased and deubiquitinating enzymes that promote the degradation of accumulated proteins are downregulated [7,19]. In the brains of Tg2576 mice, it has been reported that the ubiquitin–proteasome activity decreases with age while the Aβ levels increase [23]. In agreement with that previous study, we observed a significant increase in the global levels of ubiquitinated proteins in the hippocampus of Tg2576 mice, but not in the cortex or cerebellum. The proteins that are increasingly conjugated to ubiquitin in the hippocampus of Tg2576 mice remain unknown and to identify these was beyond the scope of our study.

Several AD-associated proteins, including APP and tau, have been reported as SUMO substrates [9,11,33]. APP can be covalently modified by SUMO-1 and SUMO-2, with these modifications resulting in decreased production of Aβ [33]. In addition, SUMO-2/3 has been variously reported to either increase [10] or decrease [20] Aβ production. Further, it has been reported that tau can be both SUMOylated and ubiquitinated, and that proteasome inhibition increased tau ubiquitination and decreased tau SUMOylation [9]. Intriguingly, a polymorphism in the non-coding region of UBC9 has been reported to predominate in AD patients, although the functional relevance of this observation was not investigated [1]. Furthermore, overexpression of UBC9 led to decreases in Aβ aggregation levels, and a localisation of UBC9 in the endoplasmic reticulum where cleavage of APP by β-secretase is thought to occur, suggesting a role of the SUMO machinery in the regulation of amyloidogenic processing of APP [33].

Changes in global levels of protein SUMOylation in the Tg2576 AD model have not been investigated previously. However, recent reports have suggested differentiated patterns of SUMO conjugation in acute cell stress, such as brain ischemia [8,31], and in diseases characterised by the aggregation of misfolded aberrant protein or polyglutamine-related pathologies (reviewed in [4,10,22]). We, and others, have previously demonstrated that SUMOylation is rapidly, dramatically and long-lastingly increased in diverse acute neurodegenerative models of cerebral ischemia [8,31]. Based on these published results, we anticipated that we would detect altered SUMOylation levels in Tg2576 mice, together with, or independent from, changes in SUMOylation-specific enzymatic machinery.

Levels of ubiquitination were robustly increased in the hippocampus with significant increases in global levels as well as for individual bands. Thus, protein ubiquitination in the vulnerable hippocampus is altered in the transgenic mice, which may reflect a dysfunction of the ubiquitin–proteasomal degradation system. In the cortex, significantly decreased levels of SUMO-2/3 conjugation were observed for distinct high molecular weight bands at ∼150 kDa and ∼130 kDa. This suggests that the SUMOylation level of these proteins may be affected and an important task will be to identify these SUMO substrates to define their relevance to AD. The significant differences seen in ubiquitin and SUMO-2/3 conjugation in the hippocampal and cortical regions are intriguing, as these regions are particularly vulnerable in the pathophysiology of AD and merit further investigation.

Total levels of the SUMO-specific conjugating enzyme, UBC9, and the dual-acting deconjugating and SUMO-maturating enzyme, SENP-1, are not altered between Tg2576 and age-matched wild-type mice. However, it is important to stress that these observations do not rule out changes in subcellular distribution or activity of these enzymes. Levels of additional components of the SUMO pathway were not explored, such as E3 ligases, which assist in substrate specificity and conjugation, and other SENP isoforms, with distinct cellular roles emerging [30].

The development of senile plaques in the brains of Tg2576 mice starts after 10 months of age, whereas the synaptic activity is reduced from 8 months [13]. So at the age we investigated (9 months) synaptic transmission is impaired but plaques are not yet evident. The mechanisms underlying synaptic function deficits in AD have yet to be fully explained but there is evidence to suggest that soluble oligomeric Aβ rather than plaques cause impaired synaptic function [5]. Surface expression of AMPA receptors, in particular the GluA1 subunit, is reduced in some transgenic APP overexpression models [3,6], which may result from changes to AMPA receptor trafficking pathways [16]. To our knowledge, levels of KA receptors have not been assessed in AD models, although the activity of these receptors is affected by Aβ *in vivo* [26]. Moreover, we reported that multiple SUMOylation targets are present at synapses and that SUMOylation can regulate the function of KA receptors [21]. The fact that we did not observe loss of either AMPA or KA receptors might be due to small changes beyond detection using immunoblotting and/or by the possibility that loss of function of these receptors results from reduced surface expression due to internalisation rather than degradation. An alternative possibility is that the deficit in synaptic transmission arises from reduced presynaptic release rather than changes in postsynaptic AMPA and/or KA receptors.

Our results suggest that global posttranslational modification of hippocampal proteins by ubiquitin and altered SUMO-2/3 conjugation in the cortex may play an important role in the mechanisms underlying AD. Identification of these ubiquitin and SUMO substrates and elucidation of their functional roles and regulation by ubiquitination and SUMOylation will undoubtedly lead to new and important insights into the pathophysiology of AD.

## Figures and Tables

**Table 1:** Comparison of individual SUMOylated or ubiquitinated protein bands in Tg2576 transgenic and age-matched wild-type mice. Discrete prominent bands were identified on the blots used for the cumulative data presented in Fig. 2 and individually compared. Arrows in the representative blots in Fig. 2 indicate the quantified band and the approximate MW values are given in the table. Data quantified from at least six animals of each group are presented as percentage of wild-type. *p*-Value indicates significance, and * on the respective blots in Fig. 2 denotes a significant difference compared to wild-type, *p* < 0.05.

SUMO-1			SUMO-2/3			Ubiquitin		
MW (kDa)	*Tg* (mean wt%)	*p* Value	MW (kDa)	*Tg* (mean wt%)	*p* Value	MW (kDa)	*Tg* (mean wt%)	*p* Value
*Hippocampus*								
Full lane	119	0.18	Full lane	137	0.18	Full lane	191	<0.01

∼130	130	0.45	∼150	94	0.86	∼100	251	<0.01
∼98	127	0.41	∼130	90	0.74	∼90	184	<0.01
∼70	115	0.58	∼85	165	0.15	∼75	176	<0.01
∼37	109	0.66	∼80	114	0.66	∼65	133	0.34
∼25	87	0.65	∼75	130	0.37	∼45	149	0.14
			∼62	156	0.16	∼40	169	0.01
			∼55	157	0.09			
			∼49	122	0.30			
			∼15	77	0.44			
*Cortex*								
Full lane	83	0.08	Full lane	89	0.48	Full lane	105	0.82

∼100	86	0.63	∼150	60	<0.05	∼130	153	0.16
∼75	62	0.35	∼130	62	<0.05	∼100	104	0.84
∼60	107	0.84	∼100	96	0.84	∼75	115	0.53
∼40	80	0.36	∼70	94	0.68	∼35	153	0.28
∼35	124	0.40	∼49	91	0.27	∼25	100	1.00
∼30	63	0.25	∼37	76	0.07			
∼27	85	0.59	∼25	81	0.26			
			∼15	130	0.28			
*Cerebellum*								
Full lane	103	0.95	Full lane	93	0.95	Full lane	94	0.74

∼100	218	0.08	∼70	112	0.72	∼130	104	0.74
∼75	126	0.69	∼69	112	0.65	∼100	86	0.52
∼60	208	0.18	∼50	111	0.72	∼85	83	0.53
∼50	105	0.92	∼40	88	0.79	∼80	89	0.66
∼30	111	0.72	∼35	129	0.70	∼70	90	0.61
						∼60	83	0.38
						∼35	79	0.45
						∼30	84	0.61
